# Impact of *KCNQ1, CDKN2A/2B, CDKAL1, HHEX, MTNR1B, SLC30A8, TCF7L2*, and *UBE2E2* on risk of developing type 2 diabetes in Thai population

**DOI:** 10.1186/s12881-018-0614-9

**Published:** 2018-06-05

**Authors:** Nattachet Plengvidhya, Chutima Chanprasert, Nalinee Chongjaroen, Pa-thai Yenchitsomanus, Mayuree Homsanit, Watip Tangjittipokin

**Affiliations:** 1 Division of Endocrinology and Metabolism, Department of Medicine, Faculty of Medicine Siriraj Hospital, Mahidol University, Bangkok, Thailand; 2 Research Division, Department of Research and Development, Faculty of Medicine Siriraj Hospital, Mahidol University, Bangkok, Thailand; 3 Department of Immunology, Faculty of Medicine Siriraj Hospital, Mahidol University, Bangkok, Thailand; 4 Siriraj Center of Research Excellence for Molecular Medicine, Department of Research and Development, Faculty of Medicine Siriraj Hospital, Mahidol University, Bangkok, Thailand; 5 Department of Preventive and Social Medicine, Faculty of Medicine Siriraj Hospital, Mahidol University, Bangkok, Thailand

**Keywords:** Type 2 diabetes, Association study, Single nucleotide polymorphisms, Thai population, Genome-wide association study

## Abstract

**Background:**

Several type 2 diabetes (T2D) susceptibility loci identified via genome-wide association studies were found to be replicated among various populations. However, the influence of these loci on T2D in Thai population is unknown. The aim of this study was to investigate the influence of eight single nucleotide polymorphisms (SNPs) reported in GWA studies on T2D and related quantitative traits in Thai population.

**Methods:**

Eight SNPs in or near the *KCNQ1*, *CDKN2A/2B, SLC30A8*, *HHEX, CDKAL1*, *TCF7L2*, *MTNR1B,* and *UBE2E2* genes were genotyped*.* A case-control association study comprising 500 Thai patients with T2D and 500 ethnically-matched control subjects was conducted. Associations between SNPs and T2D were examined by logistic regression analysis. The impact of these SNPs on quantitative traits was examined by linear regression among case and control subjects.

**Results:**

Five SNPs in *KCNQ1* (rs2237892), *CDK2A/2B* (rs108116610, *SLC30A8* (rs13266634), *TCF7L2* (rs7903146) and *MTNR1B* (rs1387153) were found to be marginally associated with risk of developing T2D, with odds ratios ranging from 1.43 to 2.02 (*p* = 0.047 to 3.0 × 10–4) with adjustments for age, sex, and body mass index. Interestingly, SNP rs13266634 of SLC30A8 gene reached statistical significance after correcting for multiple testing (*p* = 0.0003) (*p* < 0.006 after Bonferroni correction). However, no significant association was detected between *HHEX* (rs1111875), *CDKAL1* (rs7756992), or *UBE2E2* (rs7612463) and T2D. We also observed association between rs10811661 and both waist circumference and waist-hip ratio (*p* = 0.007 and *p* = 0.023, respectively). In addition, rs13266634 in *SLC30A8* was associated with glycated hemoglobin (*p* = 0.018), and rs7903146 in *TCF7L2* was associated with high-density lipoprotein cholesterol level (*p* = 0.023).

**Conclusion:**

Of the eight genes included in our analysis, significant association was observed between *KCNQ1*, *CDKN2A/2B*, *SLC30A8*, *TCF7L2*, and *MTNR1B* loci and T2D in our Thai study population. Of these, *CDKN2A/2B*, *SLC30A8*, and *TCF7L2* genes were also significantly associated with anthropometric, glycemic and lipid characteristics. Larger cohort studies and meta-analyses are needed to further confirm the effect of these variants in Thai population.

**Electronic supplementary material:**

The online version of this article (10.1186/s12881-018-0614-9) contains supplementary material, which is available to authorized users.

## Background

The prevalence of diabetes, especially type 2 diabetes (T2D), continues to increase worldwide. The International Diabetes Federation (IDF) has estimated that the prevalence of diabetes will increase from 451 million in 2017 to 693 million by 2045 [1]. The number of people with diabetes will increase as a result of changes in lifestyle caused by urbanization, high population growth, ageing population, and high economic growth. In 2017, the prevalence of diabetes among adults in Thailand was estimated to be 8.3% [1]. T2D is a complex and multifactorial disease that is characterized by impaired insulin secretion and insulin resistance [2, 3]. T2D is associated with significant morbidity and mortality, especially decreased life expectancy and reduced quality of life, as well as high cost of treatment and reduced productivity. Although the etiology of T2D is not thoroughly understood, genetic and environmental factors are thought to be involved in the pathogenesis of this disease.

Prior to the development of currently available methods and technologies, linkage and candidate gene association studies were conducted; however, these two methods could identify only a few genes involved in the pathogenesis of T2D (i.e., *PPARG, KCNJ11, CAPN10*, and *TCF7L2*). Advances in genotyping technology and genetic information have facilitated the application of genome-wide association (GWA) studies to identify T2D susceptibility genes. Currently, over 120 common risk variants have been successfully identified as being associated with T2D using this approach [4–12]. Several reproducible GWA studies confirmed these well-established susceptibility genes, and they were found to be replicated for association with T2D and diabetes-related traits in or near *SLC30A8, KCNQ1, CDC123, HNF1B, KCNJ11, TCF7L2, CDKAL1, CDKN2A/2B, PPARG, HHEX, IGF2BP2, GLIS3, JAZF1, WFS1,* and *MTNR1B* in Europeans and East Asians. Most of the susceptibility risk loci identified were shared among East Asians and populations with Europeans ancestry. However, significant association signals at these shared loci seems to be independent among populations. This suggests that the pathogenesis of the disease is common among populations, but that risk variants are often population specific. These differences in risk variants between Europeans and East Asians are likely due to differences in genetic background, risk allele frequencies, and characteristics, such as body shape, food and drink, culture, and other lifestyle factors. For example, association of variants within *KCNQ1* [6, 13] was primarily reported in GWA studies in East Asians; however, these variants were not identified in any of the larger GWA studies in European populations, because there are different frequencies of these variants between East Asian and European populations. Similarly, variants within *UBE2E2* and *C2CD4A-C2CD4B* were reported to be associated with T2D by two relatively small East Asian GWA studies, while these loci did not reach genome-wide significance in European studies [4, 5, 14]. Furthermore, there are relatively large frequency differences between population groups for rs7903146 within *TCF7L2* [15]. Although numerous T2D risk loci were identified from GWA studies in multi-ethnic populations, the functional impact of these risk loci still needs to be elucidated. Most of these risk loci likely affect insulin secretion and beta-cell function, with a few potentially involved in insulin action. Given this variability among populations, it is important that we understand the association between genetic variations and T2D, and the roles of these T2D risk loci in Thai population. In this study, we investigated single nucleotide polymorphisms (SNPs) that were previously found to be associated with T2D in Asian populations. We focused on the following eight SNPs: 1) potassium voltage-gated channel, KQT-like subfamily, member 1 (*KCNQ1*; rs2237892); 2) a variant found near cyclin-dependent kinase inhibitor 2A (*CDKN2A/2B*; rs10811661); 3) a zinc transporter member of solute carrier family 30 (*SLC30A8*; rs13266634); 4) hematopoietically-expressed homeobox (*HHEX*; rs1111875); 5) CDK5 regulatory subunit-associated protein 1-like 1 (*CDKAL1;* rs7756992); 6) melatonin receptor type 1B *(MTNR1B;* rs1387153); 7) transcription factor-7-like 2 (*TCF7L2*; rs7903146); and, 8) Ubiquitin Conjugating Enzyme E2 E2 (*UBE2E2*; rs7612463). Our specific aim was to investigate the influence of these eight SNPs on T2D and related quantitative traits in Thai population.

## Methods

### Study subjects

A total of 1000 individuals, including 500 Thai T2D patients and 500 ethnically-matched control subjects were enrolled during the 2005 to 2015 study period. T2D patients were recruited at the Siriraj Diabetes Center, Department of Endocrinology and Metabolism, Faculty of Medicine Siriraj Hospital, Mahidol University, Bangkok, Thailand. Siriraj Hospital is Thailand’s largest national tertiary referral center. Diabetes was diagnosed based on American Diabetes Association (ADA) criteria [3]. Control subjects consisted of 500 individuals without diabetes who were enrolled from the Health Check-up Center of the Department of Preventive and Social Medicine, Faculty of Medicine Siriraj Hospital, Mahidol University. Healthy controls had to satisfy all of the following inclusion criteria: age > 40 years, no family history of diabetes in any first-degree relatives, fasting plasma glucose < 100 mg/dl (6.1 mmol/l), and HbA_1c_ < 5.7%. Written informed consent was obtained from all participants after a full explanation of the study’s purpose and protocol. The study protocol and informed consent procedures were approved by the Siriraj Institutional Review Board (SIRB), Faculty of Medicine Siriraj Hospital, Mahidol University, Bangkok, Thailand (COA no. Si491/2014). This study was conducted in accordance with the principles set forth in the Declaration of Helsinki and all of its subsequent amendments.

### SNP selection and genotyping

Genomic DNA was extracted from peripheral blood leukocytes using the standard phenol-chloroform method. Eight common SNPs from eight loci were selected for analysis of association with T2D. First, we selected loci that demonstrated strong association with T2D from prior GWA studies and meta-analyses in Caucasian and Asian populations (Chinese, Japanese, and Korean). The following 8 loci were selected for investigation in this study: *KCNQ1, CDKN2A/2B, HHEX, SLC30A8, CDKAL1, UBE2E2, TCF7L2*, and *MTNR1B*. Subsequently, one SNP was selected in each locus according to the following criteria: (a) the most replicated SNP in each locus with the lowest reported *p*-value was selected; and, (b) that SNP as a tagSNP within observed Linkage Disequilibrium (LD) based on HapMap project (CHB data) using Haploview 4.2, except for SNP rs10811661 near *CDKN2A/2B*. SNP rs10811661 is out of LD mapping; however, this SNP is the most highly replicated T2D in Caucasian and Asian populations. The following 8 SNPs were tested for association with T2D: *KCNQ1* (rs2237892), *CDKN2A/2B* (rs10811661)*, CDKAL1* (rs7756992), *HHEX* (rs1111875), *MTNR1B* (rs1387153), *SLC30A8* (rs13266634), *TCF7L2* (rs7903146), and *UBE2E2* (rs7612463). Genotyping of these SNPs was performed using high-resolution melting (HRM) analysis or polymerase chain reaction-restriction fragment length polymorphism (PCR-RFLP) method (Additional file 1: Tables S2 and S3, respectively). For HRM assay, PCR was performed in 96-well plates with a total volume of 10 ul in each, which consisted of 50 ng of DNA template, 10 uM of forward and reverse primers, 1× buffer, 1.5 mM of MgCl_2_, 0.5 U of Taq DNA polymerase (Bioline, Inc., London, UK), and 1× ResoLight dye (Roche Diagnostics, Risch-Rotkreuz, Switzerland). HRM assays were performed using a LightCycler® 480 System with LightCycler® 480 Gene Scanning Software Version 1.5 (Roche Diagnostics). PCR program consisted of an initial denaturation step at 95 °C for 10 min, followed by a 50-cycle program consisting of denaturation at 95 °C for 30 s, annealing at the condition appropriate for each SNP (Additional file 1: Table S2) for 30 s, and elongation at 72 °C for 30 s, with a single acquisition mode for fluorescence signals. The melting program included denaturing at 95 °C for 30 s, annealing at 40 °C for 30 s, and subsequent melting that included a continuous fluorescent reading of fluorescence from 60 °C to 99 °C at a rate of 25 acquisitions per one degree Celcius. Quality of SNP genotyping was checked by the reproducibility of genotypes of control DNA samples, which included homozygous major allele, heterozygous major and minor alleles, homozygous minor allele, and negative control. The genotyping success rate was greater than 98% for all SNPs. Genotyping of *SLC30A8* (rs13266634) was performed by PCR, followed by restriction enzyme digestion according to the manufacturer’s instructions (Fermentas, Vilnius, Lithuania). All genotypes were confirmed by direct sequencing. Primer sequences and conditions of HRM and PCR-RFLP assays are presented in Additional file 1: Tables S2 and S3, respectively.

### Statistical analysis

Hardy-Weinberg equilibrium (HWE) was evaluated using an exact test implemented in the SNPstats online program (http://bioinfo.iconcologia.net/snpstats/start.htm). Analyses to determine associations between SNPs and T2D were performed under the additive, dominant, and recessive models using logistic regression analysis, with/without adjustment for age, sex, and BMI as covariates. In the additive model, homozygous for risk allele (1/1), heterozygotes (1/0), and homozygous for non-risk allele (0/0) were coded to a continuous variable for the genotype (2, 1, and 0), respectively. The dominant model was defined as 1/1 + 1/0 vs. 0/0, and the recessive model was defined as 1/1 vs. 1/0 + 0/0. Bonferroni test was used to correction for multiple testing. A *p* < 0.006 (0.05 divided by 8, the total number of SNPs studied) was regarded as being statistically significant. Characteristics were compared and tested for significant differences between patients and controls using Student’s t-test or Mann-Whiney U test for continuous variables, and using χ2 test for categorical variables.

Data are reported as mean ± standard deviation for quantitative variables with normally distributed data, and as median and interquartile range for non-normally distributed data. Linear regression was applied to test quantitative variables for differences between genotype groups by adjusting for age and sex for BMI; for age, sex, and BMI for weight, waist circumference, and waist-to-hip ratio; or, age, sex, and medication (as appropriate) for all other traits. Other adjustments that were made included anti-diabetic medications (e.g., sulphonylurea, metformin, and DPP4 inhibitor) for fasting plasma glucose (FPG) and glycated hemoglobin (HbA_1c_); anti-hypertensive medications (e.g., ACE inhibitors, calcium channel blocker, beta blocker) for systolic and diastolic blood pressure; and, anti-hyperlipidemic medications (e.g., statins, fibrate, and ezetimibe) for total cholesterol (TC), triglycerides (TG), low-density lipoprotein (LDL-C), and high-density lipoprotein (HDL-C). Values of FPG, HbA_1c_, TG, LDL-C, and HDL-C were natural logarithm-transformed to normal distributions before statistical analysis. Data analysis was performed using SPSS Statistics version 17.0 (SPSS, Inc., Chicago, IL, USA).

The statistical power of this study for each SNP was estimated using Quanto (http://biostats.usc.edu/Quanto.html), and powers were calculated using ORs from previously published studies, sample sizes, and minor allele frequencies (MAF). In the present study, the prevalence of T2D in Thailand was 8.3% [16], and the type 1 error rate was 0.006.

## Results

### Clinical and biochemical characteristics

The clinical characteristics of both study groups are presented in Table 1. There were significant differences between patients and control group individuals relative to age, weight, waist circumference, waist-to-hip ratio (WHR), body mass index (BMI), systolic blood pressure (SBP), diastolic blood pressure (DBP), fasting plasma glucose (FPG), glycated hemoglobin (HbA_1c_), total cholesterol (TC), triglycerides (TG), low-density lipoprotein cholesterol (LDL-C), and high-density lipoprotein cholesterol (HDL-C) (all *p* < 0.05).

**Table 1:** Demographic, anthropometric, and clinical characteristics of the control and patient study groups

Clinical characteristics	Controls (n = 500)	Patients (n = 500)	*p*-value
Gender (M, F) (%)	28.8, 71.2	32.8, 67.2	0.170164:336
Age (yrs) (years)	53.0 ± 8.4	57.2 ± 12.2	*< 0.001* ^*a*^
Age at diagnosis (yrs)	–	49.4 ± 11.3	ND
Weight (kg)	60.0 ± 10.0	67.7 ± 13.8	*< 0.001* ^*a*^
Waist circumference (cm)	83.3 ± 9.4	89.6 ± 11.4	*< 0.001* ^*a*^
Waist-to-hip ratio	0.8 ± 0.1	0.9 ± 0.1	*< 0.001* ^*a*^
BMI (kg/m^2^)	24.1 ± 3.3	27.3 ± 5.0	*< 0.001* ^*a*^
SBP (mmHg)	116.7 ± 15.4	134.9 ± 19.4	*< 0.001* ^*a*^
DBP (mmHg)	71.7 ± 10.3	79.3 ± 10.4	*< 0.001* ^*a*^
FPG (mmol/l)	3.9 (3.4, 4.4)	8.4 (6.6, 12.1)	*< 0.001* ^*b*^
HbA_1c_ (mmol/mol)	36.6 (34.2, 37.7)	57.4 (48.0, 74.9)	*< 0.001*
TC (mmol/l)	208.4 ± 37.9	200.2 ± 53.3	*< 0.001* ^*a*^
TG (mmol/l)	2.3 (2.0, 2.6)	1.5 (1.1, 2.1)	*< 0.001* ^*b*^
LDL-C (mmol/l)	2.5 (1.8, 3.5)	2.1 (1.2, 3.6)	*< 0.001* ^*b*^
HDL-C (mmol/l)	3.2 (2.6, 3.9)	1.6 (1.1, 2.2)	*< 0.001* ^*b*^

Data presented as mean ± standard deviation or median and interquartile range

A *p*-value< 0.05 indicates statistical significance

Gender distribution between patients and controls was analyzed using Pearson’s χ2 test

Comparison of quantitative variables between patients and controls was performed using Student’s t-test or Mann-Whitney U test

^*a*^*p*-values and ^*b*^*p*-values were determined using Student’s t-test and Mann-Whitney U test, respectively

*Abbreviations*: *M* male, *F* female, *BMI* body mass index, *SBP* systolic blood pressure, *DBP* diastolic blood pressure, *FPG* fasting plasma glucose, *HbA*_*1c*_ glycated hemoglobin, *TC* total cholesterol, *TG* triglycerides, *LDL-C* low-density lipoprotein cholesterol, *HDL-C* high-density lipoprotein cholesterol, *ND* not determined

### Analysis of association between SNPs and T2D

We genotyped eight SNPs in a case-control cohort of 1000 Thai subjects, including 500 T2D patients and 500 non-diabetic controls. The allele and genotype distributions of SNPs are summarized in Table 2. All 8 SNP genotypes were in Hardy-Weinberg equilibrium in the control group (all *p* > 0.05) (Additional file 1: Table S1). Logistic regression analysis was used to assess association between each SNP and T2D in 3 different genetic models (dominant, recessive, and additive). Among the 8 evaluated SNPs, the following SNPs were found to be marginally associated with increased risk of T2D after adjustment for age, sex, and BMI: *KCNQ1* (rs2237892) [odds ratio (OR): 2.02, 95% CI: 1.08–3.79; *p* = 0.020 (additive model)] and [OR: 1.43, 95% CI: 1.06–1.92; *p* = 0.018 (recessive model)]; *CDKN2A/2B* (rs10811661) [OR: 1.65, 95% CI: 1.01–2.71; *p* = 0.044 (additive model)]; *SLC30A8* (rs13266634) [OR: 1.86, 95% CI: 1.19–2.90; *p* = 0.006 (additive model)] and [OR: 1.81, 95% CI: 1.31–2.50; *p* = 0.0003 (recessive model)]; *TCF7L2* (rs7903146) [OR: 1.70, 95% CI: 1.06–2.72; *p* = 0.025 (dominant model)]; and, *MTNR1B* (rs1387153) [OR: 1.44, 95% CI: 1.01–2.08; *p* = 0.047 (recessive model)]. Interestingly, after Bonferroni correction (*p* < 0.006 indicates statistical significance), SNP rs13266634 of the *SLC30A8* gene remains significantly associated with T2D susceptibility (OR: 1.81, 95% CI: 1.31–2.50; p = 0.0003). No associations between *CDKAL1* (rs7756992) or *UBE2E2* (rs7612463) and T2D were observed in any of the 3 genetic models (Table 2).

**Table 2:** Association between eight SNPs and T2D in Thai population

Gene	SNP (major/minor alleles*)	^a^Risk allele freq.	^b^Risk allele freq.	Subject	Genotype frequency, n (%)			Additive model		Dominant model		Recessive model	
								*P* unadjusted OR (95% CI)	*P* adjusted OR (95% CI)	*P* unadjusted OR (95% CI)	*P* adjusted OR (95% CI)	*P* unadjusted OR (95% CI)	*P* adjusted OR (95% CI)
					A/A	A/B	B/B						
*KCNQ1*	rs2237892 (C > T)	0.76	0.71	Patient	285 (57.0)	192 (38.4)	23 (4.6)	0.060 1.74 (0.99–3.07)	*0.020* 2.02 (1.08–3.79)	0.128 1.53 (0.88–2.66)	0.057 1.83 (0.98–3.42)	*0.025* 1.36 (1.04–1.78)	*0.018* 1.43 (1.06–1.92)
				Control	254 (50.8)	205 (41.0)	41 (8.2)						
*CDKN2A/2B*	rs10811661 (T > C)	0.68	0.62	Patient	228 (45.6)	220 (44.0)	52 (10.4)	0.100 1.45 (0.93–2.27)	*0.044* 1.65 (1.01–2.71)	0.227 1.29 (0.85–1.96)	0.105 1.46 (0.92–2.31)	0.077 1.28 (0.97–1.69)	0.066 1.33 (0.98–1.81)
				Control	185 (37.0)	248 (49.6)	67 (13.4)						
*SLC30A8* ^c^	rs13266634 (C > T)	0.59	0.54	Patient	172 (34.4)	242 (48.4)	86 (17.2)	*0.040* 1.51 (1.01–2.21)	*0.006* 1.86 (1.19–2.90)	0.309 1.20 (0.84–1.71)	0.191 1.29 (0.87–1.91)	*0.008* 1.48 (1.11–1.98)	*0.0003* 1.81 (1.31–2.50)
				Control	137 (27.4)	268 (53.6)	95 (19.0)						
*HHEX*	rs1111875 (A > G)	0.33	0.29	Patient	234 (46.8)	200 (40.0)	66 (13.2)	*0.039* 1.61 (1.02–2.55)	0.082 1.55 (0.94–2.56)	0.260 1.16 (0.89–1.53)	0.367 1.14 (0.85–1.54)	*0.044* 1.56 (1.01–2.42)	0.087 1.51 (0.94–2.45)
				Control	254 (50.8)	201 (40.2)	45 (9.0)						
*CDKAL1*	rs7756992 (A > G)	0.43	0.40	Patient	156 (31.2)	257 (51.4)	87 (17.4)	0.249 1.27 (0.84–1.90)	0.107 1.44 (0.92–2.24)	0.355 1.14 (0.85–1.52)	0.169 1.25 (0.90–1.72)	0.340 1.19 (0.83–1.71)	0.182 1.30 (0.88–1.94)
				Control	171 (34.2)	254 (50.8)	75 (15.0)						
*TCF7L2*	rs7903146 (C > T)	0.08	0.04	Patient	429 (85.8)	67 (13.4)	4 (0.0)	NA	NA	*0.006* 1.80 (1.18–2.76)	*0.025* 1.70 (1.06–2.72)	NA	NA
				Control	456 (91.2)	44 (8.8)	0 (0.0)						
*MTNR1B*	rs1387153 (C > T)	0.44	0.42	Patient	164 (32.7)	232 (46.3)	104 (21.0)	0.113 1.35 (0.93–1.97)	0.120 1.38 (0.91–2.09)	0.618 1.07 (0.80–1.42)	0.757 1.05 (0.76–1.43)	0.058 1.38 (0.98–1.92)	*0.047* 1.44 (1.01–2.08)
				Control	174 (34.8)	231 (46.2)	95 (19.0)						
*UBE2E2*	rs7612463 (C > A)	0.83	0.82	Patient	346 (69.3)	134 (26.6)	20 (4.0)	0.875 0.94 (0.47–1.89)	0.855 0.93 (0.42–2.03)	0.890 0.95 (0.47–1.90)	0.872 0.93 (0.43–2.03)	0.847 0.97 (0.72–1.29)	0.826 0.96 (0.70–1.32)
				Control	339 (67.8)	141 (28.2)	20 (4.0)						

*Abbreviations*: *SNP* single nucleotide polymorphism, *T2D* type 2 diabetes, *Freq*frequency, *A/A* homo major allele, *A/B* heterozygote allele, *B/B* homo minor allele, *OR* odds ratio, *CI* confidence interval, *NA* not applicable (analytical model not applicable due to low frequency)

A p < 0.006 (0.05 divided by 8, the total number of SNPs studied) was regarded as being statistically significant

*P*-values were calculated for the additive, dominant, and recessive genetic models using logistic regression with/without adjustment for age, gender, and body mass index. The OR and 95% CI of having the risk allele are shown

*Alleles in bold are the risk alleles for T2D identified in previous studies, while underlined alleles indicate the risk alleles for T2D observed in this study

^a^Risk allele frequency in patients; ^b^Risk allele frequency in controls; ^c^Statistically significant after Bonferroni correction (multiple-testing) with *p*-value < 0.006

### Analysis of association between SNPs and quantitative traits

The 5 SNPs that were found to be significantly associated with T2D were then evaluated for association with quantitative traits, including body weight, waist circumference, WHR, BMI, FPG, HbA_1c_, TC, TG, LDL-C, and HDL-C using linear regression analysis after adjusting for age, sex, BMI, and medication (as appropriate) in both cases and controls (Table 3). Our analysis revealed *CDKN2A/2B* (rs10811661) to be significantly associated with both waist circumference and WHR (*p*  = 0.007 and *p* = 0.023, respectively). *TCF7L2* (rs7903146) was associated with HDL-C (*p* = 0.023), and *SLC30A8* (rs13266634) was associated with HbA_1c_ (*p* = 0.018). *KCNQ1* (rs2237892) was significantly associated at borderline with both FPG and HbA_1c_ (*p* = 0.050 and *p* = 0.052, respectively). No significant associations were observed between any SNPs and any quantitative traits in control subjects (data not shown).

**Table 3:** Association between *CDKN2A/2B* (rs10811661)*, SLC30A8* (rs13266634), and *TCF7L2* (rs7903146) genotypes and quantitative traits among Thai patients with T2D

Clinical characteristics	*CDKN2A/2B* (rs10811661) genotypes				*SLC30A8* (rs13266634) genotypes				*TCF7L2* (rs7903146) genotypes		
	T/T	T/C	C/C	*P*	C/C	C/T	T/T	*P*	C/C	C/T + TT	*P*
Number of patients	228	220	52	–	172	242	86	**–**	429	71	**–**
Weight (kg)	68.99 ± 13.96	67.42 ± 13.73	65.01 ± 14.28	0.250	66.38 ± 14.78	66.38 ± 14.78	66.38 ± 14.78	0.867	67.58 ± 13.60	68.83 ± 15.45	0.172
Waist circumference (cm)	89.34 ± 11.32	89.39 ± 11.69	92.59 ± 11.04	*0.007*	89.55 ± 11.58	89.79 ± 10.82	89.87 ± 12.75	0.506	89.66 ± 11.30	89.84 ± 12.27	0.861
Waist-to-hip ratio	0.89 ± 0.07	0.91 ± 0.08	0.92 ± 0.11	*0.023*	0.91 ± 0.07	0.90 ± 0.08	0.90 ± 0.08	0.263	0.90 ± 0.08	0.90 ± 0.080	0.334
BMI (kg/m^2^)	27.60 ± 5.06	27.07 ± 4.99	26.82 ± 5.075	0.442	26.65 ± 5.15	27.62 ± 4.72	27.59 ± 5.38	0.273	27.27 ± 5.067	27.14 ± 4.71	0.736
SBP (mmHg)	133.60 ± 19.99	134.21 ± 17.58	143.52 ± 23.29	0.746	134.94 ± 18.56	134.44 ± 19.30	136.47 ± 21.82	0.740	135.25 ± 19.64	132.98 ± 18.15	0.800
DBP (mmHg)	78.84 ± 10.72	79.42 ± 9.87	80.75 ± 11.65	0.228	79.95 ± 10.16	78.56 ± 10.85	80.18 ± 9.89	0.348	79.43 ± 10.62	78.87 ± 9.21	0.469
FPG (mmol/l)	9.27 (7.05, 12.49)	8.16 (6.38, 11.88)	7.79 (6.33, 10.68)	0.244	8.33 (6.64, 11.62)	8.49 (6.38, 12.93)	8.77 (6.84, 11.94)	0.164	8.49 (6.50, 12.25)	8.55 (6.94, 8.55)	0.721
HbA_1c_ (mmol/mol)	59.05 (49.70, 78.92)	56.30 (48.6, 71.6)	53.00 (44.82, 77.3)	0.683	56.30 (48.6, 71.6)	57.40 (49.7, 76.5)	59.05 (48.32, 78.10)	*0.018*	57.40 (48.60, 74.9)	56.30 (47.50, 56.30)	0.535
TC (mmol/l)	202.96 ± 53.19	197.52 ± 51.12	195.80 ± 60.28	0.748	197.40 ± 55.56	199.65 ± 51.20	208.33 ± 55.57	0.853	200.65 ± 54.08	198.00 ± 49.65	0.592
TG (mmol/l)	1.46 (1.03, 2.06)	1.50 (1.18, 2.11)	1.64 (1.17, 2.06)	0.197	1.43 (1.02, 2.05)	1.51 (1.14, 2.08)	1.51 (1.18, 2.26)	0.664	1.52 (1.15, 2.10)	1.32 (1.06, 1.32)	0.600
LDL-C (mmol/l)	2.22 (1.39, 3.85)	2.15 (1.30, 3.50)	1.47 (1.10, 2.87)	0.943	1.89 (1.34, 3.64)	2.38 (1.22, 3.78)	2.22 (1.37, 3.71)	0.854	2.17 (1.33, 3.68)	1.71 (1.16, 1.71)	0.741
HDL-C (mmol/l)	1.58 (1.14, 2.21)	1.63 (1.19, 2.265)	1.78 (1.44, 2.32)	0.965	1.64 (1.17, 2.24)	1.61 (1.19, 2.17)	1.55 (1.14, 2.50)	0.464	1.60 (1.16, 2.18)	1.81 (1.34, 1.81)	*0.023*

Data presented as mean ± standard deviation or median and interquartile range

*P*-values were calculated using linear regression after adjusting for age, gender, BMI, and medication, as appropriate. FPG, HbA1c, TG, LDL-C, and HDL-C values were log-transformed to improve normality before regression analysis

*Abbreviations*: *T2D* type 2 diabetes, *BMI* body mass index, *SBP* systolic blood pressure, *DBP* diastolic blood pressure, *FPG* fasting plasma glucose, *HbA*_*1c*_ glycated hemoglobin, *TC*, total cholesterol, *TG*, triglycerides, *HDL-C* high-density lipoprotein cholesterol, *LDL-C* low-density lipoprotein cholesterol

## Discussion

The present study evaluated 8 SNPs in or near the *KCNQ1*, *CDKN2A/2B, SLC30A8*, *HHEX, CDKAL1*, *TCF7L2*, *MTNR1B,* and *UBE2E2* genes for significant association with T2D. Of those, 5 SNPs [*KCNQ1* (rs2237892)*, CDKN2A/2B* (rs10811661), *SLC30A8* (rs13266634)*, TCF7L2* (rs7903146) and *MTNR1B* (rs1387153)] were found to be associated with T2D in Thai population. We also found association between *CDKN2A/2B* (rs10811661)*, SLC30A8* (rs13266634), and *TCF7L2* (rs7903146) and diabetes-related quantitative traits.

*SLC30A8* (solute carrier family 30 member 8) encodes zinc transporter-8 (ZnT8), which is specifically expressed in pancreatic endocrine cells and may participate in insulin secretion. *SLC30A8* is expressed at high levels only in the pancreas, and is highly expressed in insulin-secreting beta cells [17, 18]. Association between SNP rs13266634 in *SLC30A8* and T2D risk was discovered in European subjects [7, 9, 10]. The presence of risk C allele of rs13266634 (*SLC30A8*) was found to be associated with increased risk of developing T2D in multiethnic case-control studies, including Asians [19, 20] and Europeans [21, 22]. This SNP also showed strong association with T2D in our study with an OR of 1.78 [95% CI: 1.31–2.50; *p* = 3.0 × 10^− 4^ (recessive model)], which is higher than the odds ratios reported in previous studies (OR range: 1.1–1.2). The risk C allele frequency of 59.0% in our population was similar to frequencies reported in Asian populations (Han Chinese: 54.4%, Japanese: 52.4%), but different from European (71.7%) population (source: dbSNP database). We also found *SLC30A8* (rs13266634) to be associated with HbA_1c_ (*p* = 0.018). A similar finding was observed in a Chinese study population [23]. Functional study found that zinc transporter 8 (ZnT8) is required for normal insulin crystallization and insulin processing and secretion [24]. In addition, subjects who had homozygous of the risk C allele of *SLC30A8* showed decreased peripheral insulin levels in the early phase of intravenous glucose tolerance test (GTT) [22]. This suggests that *SLC30A8* may influence the Zn transporter, thereby affecting insulin stability and insulin secretion, but the precise role of variants of this gene remains unclear.

*TCF7L2* (Transcription Factor 7-Like 2) is a transcription factor protein that is capable of binding beta catenin, which is involved in Wnt receptor signaling. This signaling pathway affects the expression of several genes, including insulin and glucagon genes [25]. A strong association was identified between *TCF7L2* genetic variants and risk of T2D in a European population [8], which was then replicated in East Asian populations, including Chinese and Japanese [15, 26]. In this study, we found significant association between SNP rs7903146 and increased risk of T2D in the dominant model (OR: 1.80, 95% CI: 1.18–2.76; *p* = 0.006). In contrast to populations with European and African ancestries, the risk T allele of rs7903146 was rare in our Thai cohort, with a minor allele frequency (MAF) of 0.08. The *TCF7L2* gene was reported to be associated with T2D, insulin sensitivity, and insulin resistance [25]. In addition, the SNP rs7903146 was reported to be a risk factor for various metabolic disorders involving glucose and lipoprotein homeostasis [27, 28]. A previous study by our team showed significant association between an SNP in *TCF7L2* and age of onset of diabetes [29]. In that study, we found that patients who carried a minor allele of an SNP were diagnosed with T2D at an earlier age. The present study also found association between SNP rs7903146 and HDL-C among T2D patients. The specific mechanism of *TCF7L2* (rs7903146) that resides in a noncoding region and that drives the development of T2D remains unclear; however, the possible effect of the *TCF7L2* risk allele is via a defect in insulin secretion [25].

*KCNQ*1 (potassium voltage gated channel, KQT like subfamily, member 1) plays a key role in potassium channels that control insulin secretion [30]. Common variants (SNP rs2237892, rs2237895, and rs2237897) in *KCNQ1* were identified in East Asian population [6, 13]. The association of these SNPs with T2D was replicated in European, Mexican, and Asian populations [6, 13, 31, 32]. Of the three aforementioned SNPs, SNP rs2237892 appeared to have the strongest association with T2D [13]. In the present study, we also found SNP rs2237892 to be significantly associated with T2D [OR: 2.02, 95% CI: 1.08–3.79; *p* = 0.020 (additive model)] and [OR: 1.43, 95% CI: 1.06–1.92; *p* = 0.018 (recessive model)]. Other studies found variants in *KCNQ1* to be associated with impaired fasting plasma glucose, β-cell function, and metabolic traits [6, 13, 33]. However, we did not find evidence of association between *KCNQ1* and diabetic-related quantitative traits. The potential mechanism of *KCNQ1* and the pathogenesis of T2D needs to be further explored.

SNP rs10811661 is located 125 k-bases upstream from the cyclin-dependent kinase inhibitor 2A/2B (*CDKN2A/2B*) gene on chromosome 9p21. SNP rs10811661 was found to be associated with risk of T2D in multiple large GWA studies [4, 9, 10, 34]. In this study, we also found this SNP to be significantly associated with increased risk of T2D [OR: 1.65, 95% CI: 1.01–2.71; *p* = 0.044 (additive model)]. The risk T allele was found to be more common in T2D patients (MAF: 0.68), which is similar to several previous reports in Caucasian and Asian populations [19, 35–37]. In addition, the risk allele of SNP rs10811661 was associated with both waist circumference and WHR among T2D patients in this study (*p* = 0.007 and *p* = 0.032, respectively). In a similar result, a recent study from Vietnam found association between SNP rs10811661 and WHR in prediabetes subjects [38]. However, the mechanism by which the *CDKN2A/2B* locus influences diabetes risk remains uncertain. Recent publication showed *CDKN2A/2B* locus SNPs may impact T2D risk by modulating islet gene expression and beta cell proliferation [39].

Several susceptibility genes have been identified and replicated for association with T2D and diabetes-related traits in European and East Asians populations. Most of the susceptibility risk loci identified were shared among East Asians and populations of Europeans ancestry. However, a significant association signals at these shared loci seems to be independent among populations. This suggests that pathogenesis of the disease is common among populations, but that some of the risk variants may be population specific. Differences in genetic background, risk allele frequencies, and clinical characteristics, such as BMI cut-point for metabolic syndrome, diet, culture, and other lifestyle factors, may explain differences in risk variants between European and East Asians. It is, therefore, important to have information about the association of genetic variations and T2D, and to understand the roles of these T2D risk loci in different Asian populations, including Thai population. For example, rare PAX4 mutations were first identified in Thai MODY probands (designated MODY9) [40], but they are seldom found in those of European descent. A GWA study identified a susceptibility locus for T2D at 7q32 near *PAX4* [41], and exome-chip association analysis revealed an Asian-specific missense variant in *PAX4* associated with T2D in Chinese population [42]. Only one variant reached genome-wide significance (*PAX*-Arg192His, rs2233580). This association is exclusive to East Asian individuals, in whom the 192His allele is common (MAF = 10%) with a substantial effect size, with 192His observed to be virtually absent in individuals from other ancestries [43]. This suggests that *PAX4* or T2D loci may be particularly relevant in the pathogenesis of T2D in both East Asians and in Thais.

With regard to the remaining 3 loci, *HHEX* (rs1111875), *CDKAL1* (rs7756992), and *UBE2E2* (rs7612463) showed no significant association with T2D in our cohort. This result may be explained by different environmental risk profiles between Europeans and Asians, different body composition and genetic backgrounds, different linkage disequilibrium patterns, or by the fact that we have insufficient statistical power with the current sample size to replicate some of these previously reported T2D risk loci. The sample size in this study yielded power to identify significant association between T2D and variants in the *KCNQ1, CDKN2A/2B, CDKAL1, HHEX, MTNR1B, TCF7L2,* and *UBE2E2* genes ranging from 2.13 to 37.41%. A sample size ranging from 1094 to 12,600 would be needed to achieve an 80% power threshold to detect statistically significant results at alpha = 0.006. The sample size included in this study for *SLC30A8* (*n* = 500) had 96% power to detect significant association with an OR of 1.81 assuming a minor allele frequency of 0.41. This study has some mentionable limitations. First, the relatively small sample size and the commensurately limited statistical power that accompanied it may have limited our ability to identify all significant differences and associations. Second, several risk loci have been proposed as being associated with T2D in Caucasian and Asian populations. However, we limited our investigation to 8 SNPs from 8 candidate loci that were found to be common from studies in Caucasian and several different Asian populations. Thus, further studies that include the T2D risk loci not include in this study should be further investigated.

## Conclusion

Of the eight genes included in our analysis, association was observed between *KCNQ1*, *CDKN2A/2B*, *SLC30A8*, *TCF7L2*, and *MTNR1B* loci and T2D in our Thai study population. Of these, *CDKN2A/2B*, *SLC30A8*, and *TCF7L2* genes were also associated with anthropometric, glycemic and lipid characteristics. Larger cohort studies and meta-analyses are needed to further confirm the effect of these variants in Thai population.

## Additional file

Additional file 1: **Table S1** Summary of the single nucleotide polymorphisms (SNPs) investigated in this study. **Table S2** Primer sequence, PCR product size, and annealing temperature for HRM assay of designated SNPs. **Table S3** Primer sequences of SLC30A8 (rs13266634) used for PCR-RFLP method. (DOCX 27 kb)

## Abbreviations

ADA: American Diabetes Association
BMI: Body Mass index
bp: Base pair
CDKAL1: CDK5 regulatory subunit-associated protein 1-like 1
CDKN2A/2B: cyclin-dependent kinase inhibitor 2A
CI: Confidence interval
DBP: diastolic blood pressure
FPG: fasting plasma glucose
GWA: genome-wide association
HbA_1c_: glycated hemoglobin
HDL: High density lipoprotein;
HDL-C: high-density lipoprotein
HHEX: hematopoietically-expressed homeobox
HWE: Hardy-Weinberg equilibrium
IDF: International Diabetes Federation
*KCNQ1*: potassium voltage-gated channel, KQT-like subfamily, member 1
LD: Linkage Disequilibrium
LDL: Low density lipoprotein
LDL-C: low-density lipoprotein
MAF: minor allele frequencies
*MTNR1B*: melatonin receptor type 1B
ND: Non-diabetic
OR: Odd ratio
RFLP-PCR: Restriction fragment length - Polymerase Chain Reaction
SBP: systolic blood pressure
SLC30A8: zinc transporter member of solute carrier family 30
SNPs: single nucleotide polymorphisms
SPSS: Statistical Package for Social Science
T2D: type 2 diabetes
TC: Total cholesterol
*TCF7L2*: transcription factor-7-like 2
TG: Triglyceride
*UBE2E2*: Ubiquitin Conjugating Enzyme E2 E2
WHR: waist-to-hip ratio
