# Supplementary material for: Impact of KCNQ1, CDKN2A/2B, CDKAL1, HHEX, MTNR1B, SLC30A8, TCF7L2, and UBE2E2 on risk of developing type 2 diabetes in Thai population
Source: BMC Med Genet. 2018 Jun 5;19:93. doi: 10.1186/s12881-018-0614-9 (PMC5989367; doi:10.1186/s12881-018-0614-9)
Supplement: Supplementary file 1 — Table S1 Summary of the single nucleotide polymorphisms (SNPs) investigated in this study. Table S2 Primer sequence, PCR product size, and annealing temperature for HRM assay of designated SNPs. Table S3 Primer sequences of SLC30A8 (rs13266634) used for PCR-RFLP method. (DOCX 27 kb) [file 12881_2018_614_MOESM1_ESM.docx]

**Table S1.** Summary of the single nucleotide polymorphisms (SNPs) investigated in this study

| **Genes** | **Chromosomes** | **SNPs** | **Type of SNP** | **Major/minor alleles** | ***P*-value (HWE)** |
| --- | --- | --- | --- | --- | --- |
| *CDKAL1* | 11 | rs7756992 | Intronic | A>G | 0.26 |
| *CDKN2A/2B* | 9 | rs10811661 | 125kb upstream | T>C | 0.30 |
| *HHEX* | 10 | rs1111875 | Intronic | A>G | 0.59 |
| *KCNQ1* | 11 | rs2237892 | Intronic | C>T | 0.46 |
| *MTNR1B* | 11 | rs1387153 | Intronic | C>T | 0.23 |
| *SLC30A8* | 8 | rs13266634 | Missense  (Arg325Trp) | C>T | 0.09 |
| *TCF7L2* | 10 | rs7903146 | Intronic | C>T | 0.62 |
| *UBE2E2* | 3 | rs7612463 | Intronic | C>A | 0.29 |

Chi-square test was used to test for Hardy-Weinberg equilibrium (HWE) in the control group. A *p*-value<0.05 indicates that the genotype differences are not in HWE.

**Table S2.** Primer sequence, PCR product size, and annealing temperature for HRM assay of designated SNPs

| **Gene** | **SNP ID** | **Primer sequence (5’->3’)** | **PCR product size (bp)** | **Annealing temperature (ºC)** |
| --- | --- | --- | --- | --- |
| *CDKAL1* | rs7756992 | AATCAACTGCTTGCTGTTGG | 157 | 56 |
|  |  | GCTGTTCATCAGGCACTATTC |  |  |
| *CDKN2A/2B* | rs10811661 | TCTTGCCCTGTCAGCAGCT | 113 | 66 |
|  |  | GTCAAAAACCTTCCCCATCC |  |  |
| *HHEX* | rs1111875 | TCAACAGCACCATACATCATCA | 168 | 65 |
|  |  | GAAACTGCATTACTACAGACTTTCCA |  |  |
| *KCNQ1* | rs2237892 | ATGAGCCAGATGATGGGAGC | 120 | 66 |
|  |  | CCAGCCTCCAAGCTGTGTG |  |  |
| *MTNR1B* | rs1387153 | ACCATTCTCAGTGGTCCTTACTCT | 142 | 65 |
|  |  | GAAGCACAGACCTGGTTCCA |  |  |
| *TCF7L2* | rs7903146 | ACCTAGCACAGCTGTTATTTA | 227 | 52 |
|  |  | GATGAAATGTAGCAGTGAAGT |  |  |
| *UBE2E2* | rs7612463 | CAATCATTCTGCCTAATACAG | 181 | 52 |
|  |  | AAAATAGCAATAACAACAGAAG |  |  |

**Abbreviations:** PCR, polymerase chain reaction; HRM, high resolution melt; SNPs, single nucleotide polymorphisms; bp, base pair

**Table S3.** Primer sequences of *SLC30A8* (rs13266634) used for PCR-RFLP method

| **Gene** | **SNP ID** | **Primer sequence (5’->3’)** | **Product size (bp)** | **Restriction enzyme** | **Size of restriction fragment (bp)** |
| --- | --- | --- | --- | --- | --- |
| *SLC30A8* | rs13266634 | GGAGTCAGAGCAGTCGCCCA | 181 | *Alu*I | C=56+125 |
|  |  | TCGGGGTCCTGGTCAAC |  |  | T=51+56+74 |

**Abbreviations:** PCR-RFLP, polymerase chain reaction-restriction fragment length polymorphism; SNP, single nucleotide polymorphism; bp, base pair
